# COVID-19: Protecting Health-Care Workers in South Korea

**DOI:** 10.1017/dmp.2021.165

**Published:** 2021-06-07

**Authors:** Yujeong Jeon, Yeaeun Kim

**Affiliations:** 1 Department of Nursing, Koje University, Gyeongsangnam-do, South Korea; 2 Department of Health Care Management, Catholic University of Pusan, Busan, South Korea

**Keywords:** COVID-19, health-care workers, infection prevention and control

## Abstract

**Objectives:**

Health-care workers (HCWs) are vulnerable to the risk of coronavirus disease 2019 (COVID-19) infections, and the safety of HCWs is important in situations where a prolonged COVID-19 is expected.

**Methods:**

HCWs were recently put in great danger around the globe; as of December 21, there were 306 confirmed cases in HCWs in South Korea, representing 0.60% of the total 50,591 confirmed cases nationally.

**Results:**

After experiencing Middle East respiratory syndrome (MERS), South Korea has put a range of infection prevention and control (IPC) measures with long-term perspectives in place, including the use of personal protective equipment (PPE), HCW’s infection status tracing, visitor control, and a variety of supports from both national and local public health authorities.

**Conclusions:**

This article introduces the infection status of HCWs and IPC measures currently taken in South Korea, emphasizing the collaborative and long-term IPC efforts for ensuring the safety of HCWs.

As of August 3, authorities in 216 countries, areas, or territories with coronavirus disease 2019 (COVID-19) cases have reported approximately 17.6 million cases and 680,000 deaths since China reported its first cases to the World Health Organization (WHO) in December 2019.^1^ On August 4, the 14,389 confirmed cases in South Korea reached 301 deaths, and the number of patients is increasing at the time this article is being written.^2^ The consensus among health experts is that the pandemic is expected to continue spreading throughout 2020 and will remain a threat until a vaccine or effective treatment is widely available, which may not occur until the second half of 2021.^3,4^

The safety of health-care workers (HCWs) is the central issue in response to COVID-19, as well as preparation for the prolonging of COVID-19. Unfortunately, HCWs were recently put in great danger, especially when they were exposed to the risk of infection during the emergency situation at the beginning of the outbreak. A lack of knowledge on the pathogen and insufficient communication with both national and local public health authorities has increased the risk of infection for HCWs; front-line HCWs did not protect themselves effectively.

Several thousand HCWs have already been infected. In July 2020, New Amnesty revealed that more than 3000 health-care workers have died from COVID-19 in 79 countries around the world, and this figure is likely to be a significant underestimate due to under-reporting.^5^ In the early days of the COVID-19 outbreak, the Health Commission of the People’s Republic of China reported a total of 23 deaths among the 3387 HCWs infected with COVID-19 during the practice of medicine.^5^ The European Centre for Disease Prevention and Control announced that more than 37,000 of Spain’s front-line workers have been confirmed to have contracted the virus, which is the highest rate of infected health-care workers in the world, making up around 20% of all cases.^6^ In Italy, 10% of all cases were in health-care workers.^4^ As of July 5, the US Centers for Disease Control and Prevention reported that 92,572 health-care personnel had contracted COVID-19 and 507 had died as a result.^4,7^

In South Korea, since the 31st COVID-19 patient was confirmed on February 18, 2020, 4212 cases were reported by March 2 in Daegu City. Consequently, a total of 121 HCWs were infected, including 1 physician’s death.^8^ Up to this point, 71.7% of COVID-19 cases in South Korea were related to mass community infections; the largest cluster is associated with the Sincheonji Church in Daegu.^9^

As of April 5, the Ministry of Health and Welfare in South Korea officially released information on the status of HCWs infected with COVID-19. A total of 241 from 10,062 patients with COVID-19 in South Korea were HCWs (2.4%). Among them, 101 HCWs became infected while on duty.^10^

This situation prompted public health authorities to take a range of infection prevention and control (IPC) measures for ensuring the safety of HCWs. IPC strategies to prevent or limit transmission in health-care settings include ensuring triage, early recognition, and source control.^11^ As a nation, South Korea secures the supply of personal protective equipment (PPE) for anyone providing direct and indirect care to patients and traces the status of each HCW’s infection. Each local government organizes an advisory group to offer advice and training on COVID-19 for HCWs. To protect HCWs, the hospitals have complied with national IPC measures. Since the Middle East respiratory syndrome (MERS) outbreak, all tertiary hospitals have been equipped with the negative-pressure isolation rooms, and general hospitals over 300 beds have expanded the number of negative-pressure isolation rooms that meet international standards. Most hospitals have provided IPC training themselves and/or encouraged HCWs to participate in IPC training at regional and national levels. In addition to securing sufficient PPE, the preemptive use of PPE has been encouraged to the medical staff involved in front-line care. To avoid unnecessary visitor access, most entrances of hospital were closed and only some are available to visitors, who receive fever checks and are put on a visiting list. After screening COVID-19, all suspected cases are reported to local public health authorities through the national epidemiology website (ncov.mohw.go.kr), and information on the whereabouts of confirmed cases are shared with HCWs to prevent further infection. During the COVID-19 pandemic, the Ministry of Health and Welfare allowed telemedicine temporarily to minimize hospital visits. Even in the hospital, HCWs implement tele-monitoring for those being treated in negative-pressure isolation rooms.

As of July 13, Dr. Shin, a member of the Korean National Assembly, has reported 133 confirmed cases in HCWs infected from health-care settings. This represented 0.98% of the total 13,479 confirmed cases, slightly lower when compared with 1.0% by April 5 (Table 1).^12^ On December 21, 2020, the Korea Centers for Disease Control and Prevention announced that there were 306 confirmed cases in HCWs, representing 0.60% of the total 50,591 confirmed cases nationally.^13^ We can presume that the efforts mentioned above have been working properly, even if it is still too early to gauge the success in achieving the safety of HCWs.

**Table 1.** Occupational infection of HCWs in South Korea

		April 5, 2020				July 13, 2020			
Category		Total	Physician	Nursing Staff	Other HCWs	Total	Physician	Nursing Staff	Other HCWs
Number of Confirmed Cases Among HCWs	Total	101(100%)	11	82	8	133 (100%)	10	110	13
	General treatment	66(65.3%)	6	57	3	67(50.4%)	6	58	3
	Mass outbreak in the hospital	32(31.7%)	4	23	5	52(39.1%)	4	40	8
	Screening test	3(3%)	1	2	0	4(3.0%)	0	2	2
	COVID-19 treatment	0(0%)	0	0	0	10(7.5%)	0	10	0
All confirmed cases in South Korea		10,062				13,479			
HCWs‘ infection rate (%)		1.00%				0.98%			

*Note:* The number of confirmed cases among HCWs only reflects the cases in which HCWs were infected with COVID-19 during the practice of medicine, excluding community infections.

**Table 2.** IPC strategies for ensuring the safety of HCWs

Category
**Individual level**
• Keep personal hygiene • Use of emphasized PPE • Participate in IPC training
**Institutional level**
• Expand the number of negative-pressure isolation rooms • Provide IPC training and/or encourage HCWs to participate in IPC training • Supply sufficient PPE to HCWs • Restrict entrances • Screen suspected cases of COVID-19 • Notify local authorities of any suspected case • Share information on the whereabouts of confirmed cases • Implement tele-monitoring
**National public health level**
• Secure the supply of PPE and IPC equipment • Distribute PPE and IPC equipment to medical institution • Support on funding, infrastructure, and supervision for IPC measures • Allow tele-medicine temporarily • Operate an advisory group for HCWs

South Korea has learned important lessons from the MERS outbreak in 2015. At the time of the MERS outbreak, extremely crowded emergency rooms and uncontrolled visits to health-care facilities contributed significantly to nosocomial infection in some hospitals.^12^ Additionally, HCWs were placed in an extreme situation and had a high risk of MERS exposure due to vulnerable health-care environments, such as multi-bed rooms and insufficient IPC resources, including PPE and negative-pressure isolation rooms.^14^ During the MERS outbreak in South Korea, 21.1% of the 186 MERS cases occurred in HCWs, and this figure is substantially higher when compared with the current situation.^2^

While experiencing MERS, we recognized that the failure of IPC practices can lead to outbreaks among HCWs and patients. Based on the lessons from MERS, South Korea has put a variety of IPC measures for health-care facilities with long-term perspectives in place that enable the immediate and continuous launch of IPC practices. Because the implementation of IPC measures is of great importance in health-care settings, hospital-centric containment efforts have been implemented as a high priority of public health IPC, including emphasized PPE use and training at the HCW’s individual level, sufficient supply of IPC equipment and equipped facility at the health-care institutional level, active support on funding and infrastructure, and supervision at the national public health level.

In the context of an extended COVID-19 situation, keeping HCWs safe is a real concern for national public health. It is critical to make every effort in a bid not only to treat patients but also to ensure the safety of HCWs by curbing intra-hospital transmission during the progression of COVID-19. This report is meaningful in that it emphasizes that the implementing of IPC requires collaborative and long-term perspectives, both tangible and intangible measures should be supported properly and timely at all levels, as a suggestion for public health.^15^ Joint IPC measures described in this study will help HCWs stay focused on patients**’** treatments and secure their roles in hospitals by minimizing the risk of HCW’s occupational exposure.
