# COVID-19: Protecting Health-Care Workers in South Korea – ADDENDUM

**DOI:** 10.1017/dmp.2023.22

**Published:** 2023-03-03

**Authors:** Yujeong Jeon, Yeaeun Kim

The original publication of this article^1^ was missing the following acknowledgment: “This article was supported by research funds offered from Catholic University of Pusan in 2020.”
